# Assessing Overall Diet Quality: Development and Evaluation of the Performance of a Short Self-Administrated Questionnaire SCASA

**DOI:** 10.3390/nu13020677

**Published:** 2021-02-20

**Authors:** Maaike Kruseman, Angeline Chatelan, Eddy Farina, Isabelle Carrard, Jeremy Cela, Idris Guessous, Pedro Marques-Vidal

**Affiliations:** 1 Department of Nutrition and Dietetics, School of Health Sciences-Geneva (HEdS-GE), University of Applied Sciences and Arts Western Switzerland (HES-SO), Rue des Caroubiers 25, 1227 Carouge, Switzerland; angeline.chatelan@hesge.ch (A.C.); Eddy.farina.ge@gmail.com (E.F.); isabelle.carrard@hesge.ch (I.C.); Jeremy.Cela@hesge.ch (J.C.); 2 Unit of Population Epidemiology, Department of Community Medicine and Primary Care and Emergency Medicine, University Hospital of Geneva, Rue Gabrielle-Perret-Gentil 4, 1205 Geneva, Switzerland; idris.guessous@hcuge.ch; 3 Department of Medicine, Internal Medicine, Lausanne University Hospital (CHUV) and University of Lausanne, Rue du Bugnon 46, 1011 Lausanne, Switzerland; Pedro-Manuel.Marques-Vidal@chuv.ch

**Keywords:** SCASA, diet quality, dietary assessment, dietary score

## Abstract

Several tools assessing diet quality have been developed over the last decades, but their use in public health and clinical practice is limited because they necessitate detailed quantitative assessment of food intake. Our goal was to develop and validate a score (Score d’Alimentation Saine, SCASA) based on a short self-administrated online questionnaire to assess overall diet quality. SCASA targets the adult population in French-speaking Switzerland, but it was designed in a way enabling its adaptation for other regions. The choice of the items involved experts and lay volunteers. Construct validation and inter-method reliability were assessed by screening meal plans and by comparing the self-rated scores with food-record derived scores (kappa and Bland–Altman). SCASA (17 components) discriminated adequately balanced from imbalanced meal plans (93–95% and 44–46% of maximal score). Agreement between self-assessed and food record-based scores ranged between >90% (3 items), 80–89% (3 items), 70–79% (4 items), and <70% (5 items). The Bland–Altman plot showed a mean difference of −1.60 (95% CI −2.36 to −0.84), indicating a slight overestimation of the self-assessed diet quality compared to the food record. SCASA offers a reliable way to assess overall diet quality without requiring burdensome data collection or nutrient calculations.

## 1. Introduction

Diet quality plays a large role in health and disease [1]. Despite some controversies, the evidence-based dietary priorities related to the prevention of several major chronic diseases are widely agreed on: they include the increased consumption of vegetables, fruits, whole grains, legumes, and nuts; the wise choice of oils; and the decrease of (red) meat and highly processed foods [2,3,4,5,6]. Public health policies, health promotion programs and (primary or secondary) prevention practices targeting individuals share the goal of reducing the burden of diet-related conditions. The evaluation of their efficacy on diet mostly relies on dietary assessments [7,8,9,10,11,12,13]. After decades of focusing on nutrient intake, a practice inherited from an era where food shortage and nutritional deficiencies were a major threat [2], the emphasis has shifted toward the assessment of the whole diet [6,14,15]. Since the late 1990s, nutritional epidemiologists have warned against the evaluation of diet quality based solely on nutrient intake, which can hide paradoxical situations, for example when nutritional requirements are fulfilled by excessive intake of ultra-processed foods [16,17,18].

Over the years, several tools have been developed to assess the overall quality of the diet with a comprehensive score, such as the healthy eating index (HEI) [19,20] and its alternatives [8], the Mediterranean diet score [21,22], the French Programme National Nutrition Santé Guidelines Score (PNNS guidelines score) [23] or the Nordic nutrition recommendations score [24]. These tools define good diet quality as the compliance with relevant dietary guidelines [20,23,24], and better scores have been associated with a lower risk of weight gain [25,26], cardiovascular diseases [8,9,27,28], type 2 diabetes [8,28,29], several cancers [7,9], and mortality [9,22,28,30].

Despite the advantage of assessing diet quality as a whole, three characteristics of these tools can limit their use. First, they necessitate the detailed quantitative assessment of food intake using extensive food questionnaires, such as a >100-item food frequency questionnaire or several 24-h dietary recalls conducted by nutrition professionals. These dietary assessment methods put a high burden on study participants and require specific expertise for the data collection and analyses, which necessitates substantial financial resources [31]. Second, the computation of these scores requires information on daily nutrient intake. Total food intake must therefore be assessed accurately, and each food item must be linked with an appropriate nutrient database, which again increases the burden on participants and investigators. Moreover, it has the potential of biasing the total score, by attributing points according to nutrient intake regardless of their source. The Nordic nutrition recommendations score [24], for example, relies solely on nutritional intake, and could favor over-consumers, including those with less favorable diets [32]. Accounting for total energy intake (which is the case for the HEI-2010) does not completely cancel this bias, because large consumers of (ultra-) processed foods, especially when these are fortified, might also be favored in terms of vitamin and mineral intake. Another illustration is pizza, grain-based desserts, chicken- and fish-based mixed dishes that are among the main sources of mono- and poly-unsaturated fatty acids in the US, despite the guidelines promoting vegetable oil, nuts and seeds, and unprocessed fish to fulfil nutritional requirements in unsaturated fat [33,34]. Third, due to complex data management, immediate feedback about the score results cannot be provided to individuals, whereas feedback is sought-after in clinical and health promotion contexts, and could trigger constructive discussions about nutrition between health professionals and their patients [35].

In response to these limitations, researchers have developed short food-based screening tools to characterize individuals’ diet quality in studies with limited resources, and to enable non-nutritionally trained personnel to rapidly estimate individuals’ dietary patterns. Classical examples are the Mediterranean diet adherence screener in Spain (MEDAS) [36,37], the diet quality tool in Australia [38], and the SmartDiet in Norway [39] and Canada [40]. These self-administrated scores, designed to provide immediate feedback to individuals, evaluate the overall diet quality by focusing on 9 to 15 foods or food groups, and assessing the adherence to the local dietary guidelines [36,37,38,39,40]. These tools, albeit validated, cannot be transposed easily into a different population because their content (e.g., the foods they are based on) is highly specific to the country in which they have been developed, or to the health-related factor under focus.

To our knowledge, no such score exists in Switzerland. Therefore, our goal was to develop a tool based on guidelines that are compatible with those of other countries, and to validate it in the French-speaking part of Switzerland. More specifically, our objectives were: (1) to develop a score based on a self-administrated online questionnaire to rapidly assess overall diet quality for the prevention of diet-related chronic diseases; (2) to evaluate the score’s ability to screen individuals according to their eating patterns.

## 2. Materials and Methods

**Figure 1:**
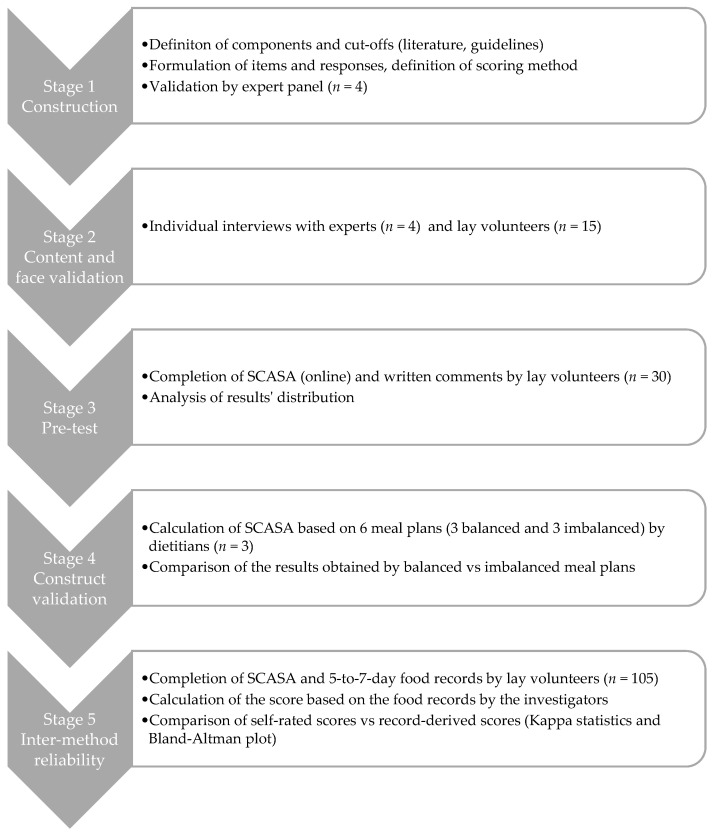
Overview of the development process of SCASA (Score d’Alimentation Saine).

SCASA (score d’alimentation saine, or healthy eating score in French) targets primarily the adult population in French-speaking Switzerland, but we designed it in a way that enables its adaptation and use in other regions or countries (Appendix A). The development of the score involved a multistage process (Figure 1), with adjustments after each stage according to the outcomes. The Geneva Cantonal Ethics Committee on Research Involving Humans reviewed and approved this study (Project 73,457). All participants signed an informed consent form.

### 2.1. Stage 1: Construction of SCASA

The item selection (i.e., score components), their cut-off values, and the scoring method are key steps in the construction of a diet quality score [41]. To construct SCASA, we used the RAND/UCLA appropriateness method, a guided process for medical decision making in the face of limited evidence [42,43]. First, we identified the potential components to include (e.g., consumption of fruits, consumption of meat, physical activity) based on a review of existing scores [41,44] and the national food-based Swiss dietary guidelines [45,46].

We submitted the list of potential components (*n* = 19) with their definitions (i.e., included foods and their impact on health) to a panel of four national experts in nutritional epidemiology and public health. The experts expressed their opinion about the appropriateness of each component in two rounds: the first individually, the second during a panel meeting moderated by a person not involved in the project. During the panel meeting, which was audio-recorded and transcribed, the experts discussed the relevance of each potential component and voted for its (non-)inclusion in the score. Five components were excluded (caloric beverages, fruit juice, candy, processed food, food variety) and two were split into several distinct items (starchy foods and whole grains, all meat and red meat). Corpulence was added as a component.

For the chosen 17 components, we defined the cut-offs based on international [47,48] and national [45,46] dietary guidelines, as well as national population-based food consumption data [49,50]. Sixteen components were evaluated in terms of quantity and/or frequency (e.g., “3 portions of 120 g a day”, “up to 3 times a week”), one component (i.e., type of fats used) was assessed qualitatively, and one component characterized corpulence as a proxy for energy intake. We then phrased the items (i.e., questions, possible answers, and examples of included foods and portion sizes) and established the scoring method. In this first stage, corpulence was categorized using either body mass index categories or the Lorentz formula [51], in order to test both models. Following the procedure described by Estaquio et al. [23], we established a two-to-four-point scale within items: +2 or +1 (guideline fully respected), 0 (guideline partially respected), and −1 (guideline not respected). Each item had the same weight in the overall score computation. Total score was expressed as a proportion of the maximal score.

### 2.2. Stage 2: Content and Face Validation

We interviewed each of the four experts individually, asking them to review the items, cut-offs and scoring method, which led to refining the wording of some items but no major changes. We followed the suggestion of one expert to include a rating of self-perceived diet quality on a Likert scale, and an estimation of physical activity level for descriptive purposes (not included in the score calculation).

We recruited by word of mouth 15 volunteers with various characteristics, in terms of age, gender, socioeconomic status, and corpulence, and without specific nutritional knowledge. During an individual, semi-directed interview of approximately 45 min, each individual completed a SCASA on paper, during which they explained aloud their understanding of each item and their reflections about how to answer the questions. Each interview was audio-recorded. This stage led to the simplification of the questionnaire’s introduction, the modification of several items’ wording, and more precise examples of portion sizes (e.g., for vegetables). The corrected version of SCASA was then put online on a secure platform (EvaSys, Stat’Elite, Yens, France, version 7.1).

### 2.3. Stage 3: Pre-Test and Internal Consistency Assessment

This first version of SCASA was pre-tested in a sample of 30 volunteers recruited by an email sent to all second-year bachelor students at the Geneva School of Health Sciences (nursing, midwifery, nutrition and dietetics, physiotherapy, and radiology technology). The students were asked to fill the questionnaire online and provide written comments regarding the completion process. We analyzed the distribution of the responses to each item and of the total score in order to detect a ceiling or floor effect (i.e., grouped responses at the top or bottom of the distribution, respectively) or unclear questions leading to numerous missing answers and comments from students. One item (i.e., candy) was removed after this stage, leaving the final score with 17 items, for a score ranging from −19 to 19.

### 2.4. Stage 4: Construct Validation

We modelled the process of the construct validation proposed by Guenther et al. for the HEI [20] by assessing the ability of the score to discriminate balanced from imbalanced meal plans (Appendix B). The investigators (i.e., dietitians), created six weekly meal plans: three balanced plans according to the Swiss dietary guidelines [45,46,47], and three imbalanced plans: (1) western-type diet rich in ultra-processed foods [52,53]; (2) low-carb western-type diet rich in protein-based foods; (3) very low caloric weight loss diet. Out of the six meal plans, two were vegetarian (one balanced and one imbalanced). Three experienced dietitians external to the project reviewed all six meal plans, and three other dietitians, blinded to the project, completed SCASA for each meal plan (Appendix B). The average score of each plan was compared with the maximum score obtainable. Our hypothesis was that the balanced and imbalanced meal plans would obtain ≥80% and ≤30% of the maximum score, respectively.

### 2.5. Stage 5: Inter-Method Reliability Assessment

**Figure 2:**
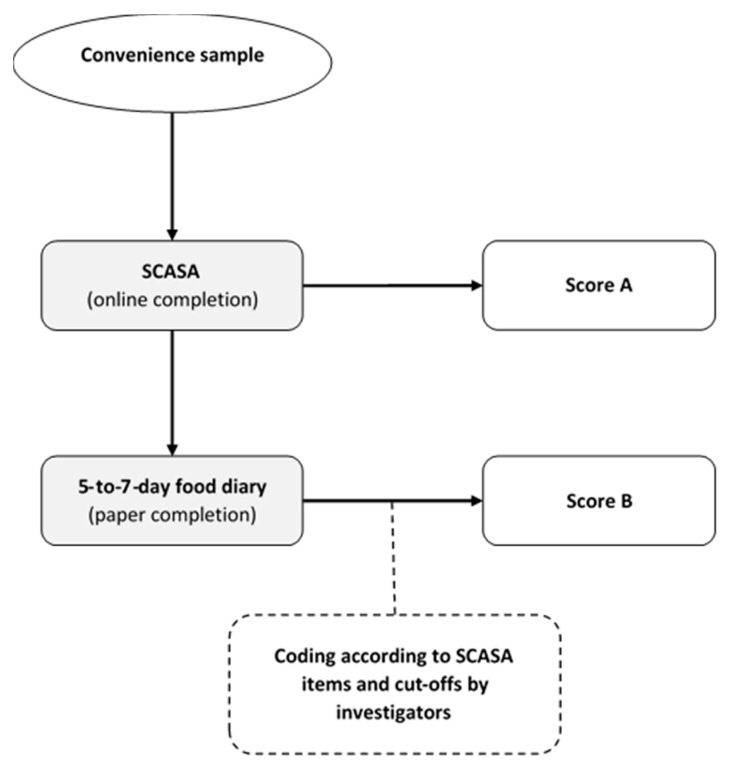
Inter-method reliability assessment of SCASA (score d’alimentation saine).

In order to assess the reliability of SCASA, we evaluated the concordance of the scores obtained by self-rating (i.e., when an individual fills the questionnaire) with the scores obtained with a reference method (i.e., calculation by nutrition experts on the basis of a 5-to-7-day food record) (Figure 2). Food records were considered preferable to repeated 24-h dietary recalls because they can capture the weekly consumption of foods that are not consumed very often. Albeit burdensome, food records also provide more accurate and precise information on food consumption than a food-frequency questionnaire (FFQ) [40,54,55,56].

For this inter-method reliability assessment, we recruited a sample of 105 volunteers from the target population (73% women, mean age 30 ± 13.7), who completed SCASA online (no feedback provided). One week after completion, they received oral (by phone) and written instructions (by email) on how to fill in a paper-based food record. To analyze the food records we grouped the reported food items according to the items defined by SCASA, and assigned points to each record-derived item using the same cut-offs as in SCASA. We assessed agreement between each self-rated and record-derived item using quadratic weighted kappa statistics. Agreements were classified as follows: weak <70%; fair 70–79%; moderate 80–89%; and strong ≥90%. The participants did not report consistently the type of oils and fats in their food record, and therefore the item “Fats and oils” could not be included in the quadratic weighted kappa statistics. The item “Corpulence” was also not included in the food record. We then assessed agreement between the total score obtained with SCASA and with the food record using Bland–Altman plots. Limits of agreement were set at 1.96 × standard deviations above and below the mean difference [36,40,57]. As the goal of SCASA is to assess the overall quality of the diet and not to assess nutrient intake, we chose not to show nutritional intake data.

### 2.6. Adaptations for Other Regions

SCASA is mainly based on the Swiss national dietary guidelines [45,46]. In order to facilitate the adaptation for other countries, we have tabulated the recommendations next to those of Great Britain, France, Germany, Belgium, and the Netherlands in Appendix A.

## 3. Results

### 3.1. Description of SCASA

Table 1 describes the 17 components, cut-offs, and scoring method of SCASA after content and face validation, and pre-testing. In addition to these items, the respondents were asked to give a general estimation of their diet quality (Likert scale from 0 to 10) and physical activity level (sedentary, light, active, and very active), and to state their sex, age, weight, and height. It takes 15 to 20 min to fill in the online version of SCASA.

### 3.2. Construct Validation

The balanced (*n* = 3) and imbalanced (*n* = 3) weekly meal plans obtained, respectively, 93 to 95% and 44 to 46% of the maximal score, showing a good discrimination between balanced and imbalanced dietary patterns.

### 3.3. Inter-Method Reliability

The agreement between self-assessed and food-record based SCASA-scores for each item is shown in Table 2. Agreement was considered as strong for three items: “Vegetables”, “Starchy foods”, and “Alcoholic beverages”. Mismatches (low agreement and kappa value <0.2) between the self-assessed and food-record based scores were observed for two items in particular “Total meat” and “Nuts and seeds”.

**Figure 3:**
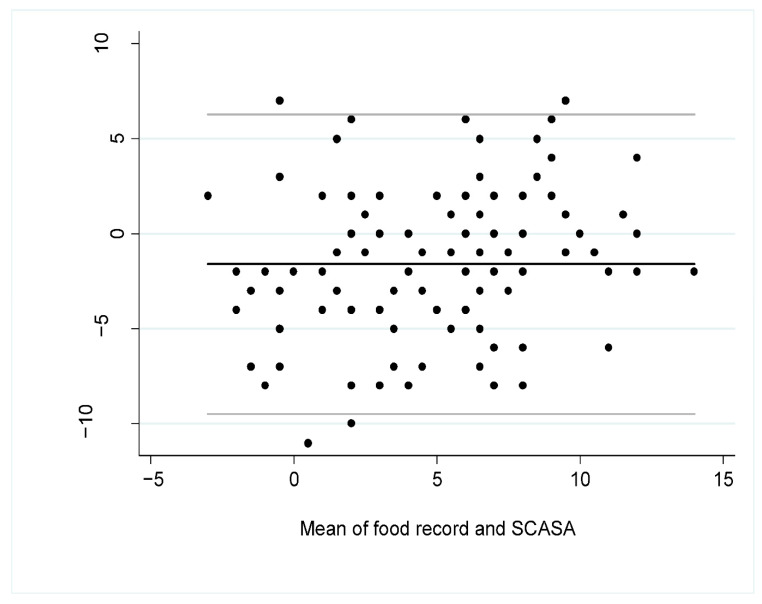
Bland–Altman plot for overall agreement between the total score obtained with SCASA (Score d’Alimentation Saine) and with the 5-to-7-dayfood record (i.e., reference method) (*n* = 105). The black line represents the mean bias between the two measures and the grey lines are the 95% limits of agreement.

The Bland–Altman plot (Figure 3) showed that the mean difference of the scores according to the assessment method was −1.60 (95% CI −2.36 to −0.84), indicating a slight overestimation of the diet quality with the self-assessment (SCASA) compared to the food record, especially when the diet was of lower quality, i.e., when the total score was closer to negative values (Figure 3).

## 4. Discussion

Our goal was to develop a score assessing the overall diet quality of Swiss adults (SCASA).

SCASA discriminated adequately between healthy and unhealthy diets, as shown by the consistent results obtained when applying the score to optimal and suboptimal meal plans. The imbalanced meal plans obtained higher scores than hypothesized (45% of maximum score obtainable vs. 30%). These rather generous scores can be explained by the fact that SCASA does not consider foods as “unhealthy” per se, but penalizes only very inadequate consumption. Another explanation is that the “unhealthy” food items were grouped into four items (i.e., sweets, salty snacks, and fatty dishes representing one item), whereas the “healthy” foods were detailed into 12 items, resulting in a lower influence of unhealthy foods on the final score.

Reliability of SCASA was fair, similarly to other instruments, such as the recently developed short healthy eating index survey (sHEI) which shows correlations with a 24 h recall score ranging from 0.44 to 0.64 for individual food group items [58]. Meat consumption was underestimated by SCASA compared to the food records. This might be explained by the fact that any consumption of meat reported in the food records, regardless of quantity, was taken into account, whereas the respondents might not have counted the very small quantities of meat in preparations when assessing their overall weekly intake. For example, when consuming a mixed salad with small pieces of salami, a respondent might not count this as an occasion of meat consumption, whereas it will be counted during the rating based on their food record. The consumption of nuts and seeds was either overestimated (e.g., when the participants counted salted peanuts in this category whereas it belonged to the “Snacks” item), or underestimated (when the participants did not count the nuts present in composed dishes or as ingredients). This observation led us to clarify the definition of the item in the final version. The fair agreement obtained by the item “Whole grains” can be explained by their infrequent consumption, and that for “Red meat” could be related to a temporal bias. Indeed, the food records of the discrepant pairs showed an increased consumption of grilled meat, related to the barbecue-favorable weather that appeared between the two assessments.

Two items “Starchy foods” and “Alcoholic beverages” presented a kappa paradox (i.e., a low kappa despite excellent agreement). Indeed, kappa tests are less reliable when tables are symmetrically imbalanced, i.e., when most people rate themselves similarly [59]. This was the case for starchy foods, which most participants reported eating at least twice a day.

The limitations of SCASA are those that are inherent to dietary assessment. Very strong agreement is rare when comparing dietary assessments methods, because of the complexity of the diet (large number of foods available, variability over weekdays and seasons, variation in nutrient composition of similar foods and recipes) and the related difficulties of self-assessing diet [60,61]. Food records, although considered as the reference method, are subject to measurement errors, notably because they reflect consumption during a short time frame. We overcame some of these limitations by combining several validation methods.

SCASA offers a complementary tool to those already existing. Similarly to the sHEI [58], it does not require burdensome data collection or nutrient calculation, and focuses solely on overall, food-based, diet quality. The authors of the sHEI noted that some of their questions might have been difficult to understand for the respondents (i.e., “How many servings of saturated fat do you consume on average per day?”) [58]. The strength of SCASA is that is has been tested among a panel of lay people, which increased its intelligibility and hence the reliability of the answers. Communication about respondents’ current diet is key to improve awareness and trigger change [33]. As part of the implementation process, feedback on total score, fats and oils, plant-based foods, meat, dairy products and other sources of calcium, snacks and sweets, and alcoholic beverages was developed and tested. The texts, following a structure inspired by the health belief model [59] are in French and may be obtained from the authors upon request.

## 5. Conclusions

SCASA offers a reliable way to assess overall diet quality without requiring burdensome data collection or nutrient calculations in the French-speaking part of Switzerland, with the possibility to adapt it to other regions in Western Europe.

## Figures and Tables

**Table 1:** Description of SCASA (Score d’Alimentation Saine) items and scoring method.

#	Components	Types of Assessment (Portion of Reference)	Answers (i.e., Cut-Offs)	**Scoring**
1	Fruits (excluding juices)	Daily quantity (≅100 g)	<1	−1
			1	0
			2	+1
			>2	0
2	Vegetables (excluding potatoes)	Daily quantity (≅120 g)	≤1	−1
			2	0
			3	+1
			>3	+2
3	Starchy foods (e.g., bread, pasta, rice, potatoes)	Daily frequency	<1	−1
			1	0
			≥2	+1
4	Whole grains (e.g., whole-grain bread, brown rice)	Weekly frequency	<1	−1
			1	0
			≥2	+1
5	Legumes (e.g., beans, lentils, chickpeas)	Weekly frequency	<1	−1
			1–2	0
			>2	+1
6	Cheese and other dairy products	Daily portion number Examples of portions provided in questionnaire, 200–250 mg calcium/portion and an average of 10 g protein. Two portions of dairy products account for one extra portion of protein-based foods in the score calculation.	≤1	−1
			2	0
			3	+1
			≥4	−1
7	Protein-rich foods (plant or animal based) (e.g., meat, fish, seafood, eggs, legumes, tofu)	Daily portion number Examples of portions provided in questionnaire, ≅20 g protein/portion.	<1	−1
			1–2	+1
			>2	−1
8	Total meat (including processed meat)	Weekly frequency	0–3	+1
			3–4	0
			>4	−1
9	Red meat (e.g., beef, veal, pork, lamb, horse)	Weekly frequency	Never or almost never	+1
			≤2	0
			>2	−1
10	Processed fatty meat (e.g., sausages, cold cuts)	Weekly frequency	Never	+1
			Rarely, up to 1	0
			>1	−1
11	Fish and seafood (including canned and smoked fish)	Weekly frequency	Never or almost never	−1
			<1	0
			≥1	+1
12	Fats and oils used for cooking (hot) or seasoning (cold)	Types of fats A list of fats and oils is provided	Used for cooking	
			HOLL * rapeseed, HO ** sunflower, refined olive, peanut oils	+1
			Refined rapeseed, sunflower, safflower, soya oils	0
			Extra-virgin olive, flaxseed, hazelnut, walnut oils, frying fat, coconut fat, margarine, butter	−1
			Used for seasoning	
			Refined rapeseed, Extra-virgin olive refined olive, flaxseed, hazelnut, walnut oils	+1
			HOLL * rapeseed, (HO **) sunflower, peanut, safflower, soya oils, butter	0
			frying fat, coconut fat, margarine	−1
13	Sweets and salty snacks, high fat dishes and sauces (e.g., pastries, cream-based desserts, biscuits, chocolate, chips, cheese pies, French fries, fried spring rolls, pesto, cream sauce)	Weekly frequency	0–14	+1
			15–21	0
			>21	−1
14	Nuts and seeds (e.g., avocado, almonds, olives, sunflower seeds)	Weekly/daily quantity Examples provided: 20 g almonds or nuts, ¼ avocado	<2/week	−1
			2–6/week	0
			1/day	+1
			>1/day	−1
15	Sugar-sweetened beverages (e.g., soft drinks, ice tea, fruit juices and lemonades, milk-based sugary drinks, sport and energy drinks, excluding those with artificial sweeteners)	Weekly quantity	0–1 L	+1
			>1 L	−1
16	Alcoholic beverages	Weekly quantity Examples of portions provided in questionnaire, 1 unit alcohol/portion	Men	
			<15	+1
			≥15	−1
			Women	
			<10	+1
			≥10	−1
17	Corpulence	% calculated weight according to Lorentz formula	80–120%	+1
			120–130%	−1
			>130%	−2
			<80%	−1

* HOLL: high oleic, low linolenic fatty acids content, ** HO: high oleic fatty acids content.

**Table 2:** Agreement between self-assessed and food-record based SCASA scores.

#	Components	Observed Agreement between Two Ratings	Quadratic Weighted Kappa Value	Comments	Overall Assessment of Agreement
1	Fruits	76%	0.37	-	Fair
2	Vegetables	90%	0.33	-	Strong
3	Starchy foods	94%	−0.02	Kappa paradox due to symmetrically imbalanced table	Strong
4	Whole grains	67%	0.24	-	Weak
5	Legumes	83%	0.33	-	Moderate
6	Cheese and other dairy products	69%	0.20	-	Weak
7	Protein-rich foods	68%	0.21	-	Weak
8	Total meat	65%	0.18	-	Weak
9	Red meat	79%	0.21	-	Fair
10	Processed fatty meat	73%	0.21	-	Fair
11	Fish and seafood	84%	0.42	-	Moderate
12	Fats and oils used for cooking (hot) or seasoning (cold)	-	-	Not assessed in food record	-
13	Sweets and salty snacks, high fat dishes and sauces	81%	0.16	-	Moderate
14	Nuts and seeds	66%	0.14	-	Weak
15	Sugar-sweetened beverages	74%	0.36	-	Fair
16	Alcoholic beverages	98%	0.49	Kappa paradox due to symmetrically imbalanced table	Strong
17	Corpulence	-	-	Not assessed in food record	-

**Figure A1:**
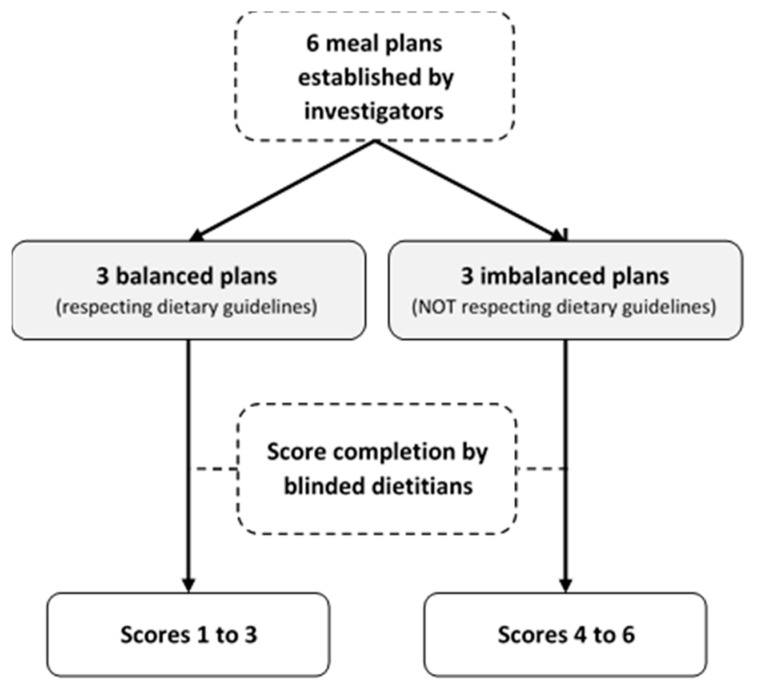
Construct validation process of SCASA (Score d’Alimentation Saine).

## Data Availability

Data available on request from the corresponding author due to privacy and ethical restrictions.

## Appendix A

**Table A1:** Comparison of the dietary guidelines in various countries for the components used in SCASA (Score d’Alimentation Saine).

Component	Switzerland [62] *	Great Britain [63]	France [64]	Germany [65]	The Netherlands [66]	Belgium [67]
Fruits	2 servings/day	5 servings/day	2 servings/day	2 servings/day	200 g/day	250 g/day
Vegetables	3 servings/day		3 servings/day	3 servings/day	200 g/day	300 g/day
Starchy foods	3 servings/day	Every meal	Every meal	Every meal	-	Sufficient quantity every day
Whole grains	Promote	Promote	Promote	Promote	At least 90 g/day	At least 125g/day
Legumes	Promote	Consume more	2 servings/week	-	Every week	At least 1 portion/week
Cheese and other dairy products	3 servings/day	Every day	2 servings/day	200–250g of milk and dairy products and two slices of cheese (50–60 g) per day	A few servings per day	250–500 mL of milk and dairy products per day
Protein-rich foods	1 serving/day. Vary between meat, fish, eggs, quorn, seitan, or cheese	Vary the sources of protein	Vary the sources of protein. Choose poultry and limit other meats	300–600 g of meat, 2 servings of fish and 3 eggs per week	Max. 500 g/week of meat	Vary the sources of protein. 1–3 servings/week poultry, egg or other meat substitutes
Total meat	2–3 servings/week	-	500 g/week maximum	300–600 g/week	-	-
Red meat	-	-	-	-	300 g/week maximum	300 g/week maximum
Processed fatty meat	1 serving/week	70 g/day maximum	150 g/week	-	-	30 g/day maximum
Fish and seafood	-	2 servings/week including 1 of fatty fish	2 servings/week including 1 of fatty fish	1–2 servings/week	1 serving/week, preferably fatty fish	1–2 servings/week including 1 of fatty fish
Fats and oils	2–3 tablespoons (20–30 g) of vegetable oil per day, at least half of which is rapeseed oil Small amount of butter, margarine, cream can be consumed every day	Unsaturated oils and small amounts	Rapeseed, walnut and olive oil. Added fats (oil, butter and margarine) can be consumed daily in small amounts	10–15 g of oil (rapeseed, walnut or soybean oil) and 15–30 g of margarine or butter. Prefer vegetable oils and especially rapeseed oil		Rapeseed, soy, and walnut oils
Sweets and salty snacks, high fat dishes and sauces	1 small portion per day maximum	Reduce, consume occasionally and in small quantities	Limit their consumption	Not recommended		Limit sweet products
Nuts and seeds (unsalted, without sweet coating)	1 serving (20–30 g)/day	-	-	-	≥15 g/day	15–25 g/day
Sugar-sweetened beverages	Limit their consumption	-	Limit as much as possible. 1 serving/day maximum	-	-	-
Alcoholic beverages	Men: 2 serving/day maximum Women: 1 serving/day maximum	-	2 servings/day maximum, not every day	-	1 serving/day maximum	Men: <20 g/day (two servings) Women: <10 g/day (one serving)

* For alcoholic beverages, recommendations of the national center for addiction prevention (addiction suisse) apply.
